# The Cognitive Cost of Repetitive Thinking: A Study on the Effects of Shifting and Updating on Rumination of Emotional Experiences

**DOI:** 10.3390/brainsci13111569

**Published:** 2023-11-09

**Authors:** Fabiana Battista, Tiziana Lanciano, Patrizia Borrelli, Antonietta Curci

**Affiliations:** Department of Education, Psychology, Communication, University of Bari ‘Aldo Moro’, 70121 Bari, Italy; tiziana.lanciano@uniba.it (T.L.); patrizia.borrelli@uniba.it (P.B.); antonietta.curci@uniba.it (A.C.)

**Keywords:** working memory, updating, shifting, rumination

## Abstract

The present study aimed to investigate the consequence of resource competition between post-emotional processing and concurrent cognitive tasks. Previous studies have shown that such a resource competition engenders both short-term (e.g., defeats in the execution of the working memory task) and long-term effects (e.g., procrastination or rumination following an emotional experience). We expected these effects to vary as a function of the different WM components involved (shifting, Study 1; updating, Study 2). In two studies, participants (Study 1: *N* = 48; Study 2: *N* = 42) were administered one out of two variants of a visuospatial task (Study 1: shifting; Study 2: updating) adopted by Curci and colleagues before and after a negative or neutral manipulation. Rumination was assessed immediately after the second WM task performance and 24 h later. In Study 1, results showed that the exposure to negative content impaired the subsequent executive performance compared with exposure to neutral material, while no difference was found in Study 2. Rumination for emotional material was higher and more persistent over time as a function of shifting resources but not for updating ones. These findings provide information on the possible role of individuals’ cognitive resources on rumination for emotional experiences.

## 1. Introduction

A large amount of research has demonstrated that experiencing a negative event may result in repetitive thoughts concerning such an experience [1,2,3,4,5]. The repetitive thinking occurring after the negative experience is a cognitive elaboration of the experience and occurs in different forms [5]. One of these forms is rumination, which was defined as “a class of conscious thoughts that evolve around a common instrumental theme and that recur in the absence of immediate environmental demands requiring the thoughts” [6] (p. 7). Survey studies have also displayed that around 80% of the population who experienced a negative event have a persistency of ruminative thoughts for long periods of time ranging from 4 to 23 years [7,8]. Although rumination has been widely studied as an individual’s response to regulate traumatic or negative experiences (i.e., depressive rumination) (see the Response Style Theory; RST) [9], rumination can, in turn, manifest for other circumstances [10,11].

So far, several studies have confirmed Horowitz’s idea [12] that rumination, framed as an ordinary, prolonged, disturbing, and difficult-to-stop cognitive process due to an emotional negative impact, requires a cognitive effort in order to try to manage the perturbing and persistent thoughts and this results in a competition of cognitive resources [2,3,13,14]. Hence, these studies suggested a strong relationship between rumination and cognitive processes and functioning.

### 1.1. Rumination and Working Memory

In 2013, in his working memory (WM) model, Baddeley [15] assumed that rumination occurs because of a malfunctioning of the central episodic buffer of WM. Subsequently, a high number of studies supporting this view were conducted, mainly regarding depressive rumination and clinical samples. Only a few studies have tried to understand whether repetitive thinking of an emotional event as a post-experience process equally resulted in poor performance at a cognitive task in normal populations [2,16,17,18].

Curci and colleagues [2], for instance, conducted a study to test the influence of rumination for a negative experience on concomitant performance in a working memory task. In addition, the authors were also interested in investigating whether the individuals’ availability of WM resources further explained this influence. They asked participants to perform a WM task before and after the presentation of either a negative or neutral narrative. Then, participants filled in a series of questionnaires to report their ruminative thoughts about the narrative, both immediately after the second WM task and after 24 h. Participants were also split into two groups based on their WM availability (i.e., low vs. high WM resources). The authors found that individuals’ WM availability and the valence of the narrative influenced the performance at the WM task and ruminative thoughts, such that low WM resources had a worse performance and higher ruminative thoughts than high WM resources, especially if they read the negative excerpt. Interestingly, rumination mediated the association between the negative state induced and the subsequent WM performance. Collectively, these findings suggested that rumination detrimentally affects WM resources, and, in turn, this makes rumination last longer.

After this attempt, Curci and colleagues [3] conducted two additional experiments with the aim of demonstrating that the cognitive resources competition between the emotional experience processing and the performance of a cognitive task (i.e., WM) causes a worse performance at the WM task and a prolongation of ruminative thoughts over time. They also detected whether this competition would affect the formation of intrusions (i.e., visual images and sensory impressions of the emotional event). In both studies, they adopted a similar procedure as a study carried out in 2013 [2]. However, in their first experiment, they proposed participants perform either a verbal measure of WM or a visuospatial measure of WM, while in their second study, they asked participants to either read an emotional narrative or watch a negative video clip. They added these levels in their designs to compare the “modality” hypothesis (i.e., sensory/visuospatial encoding of traumatic experiences increases the frequency of later ruminative thoughts) [19,20] with the “distraction” hypothesis (i.e., any activity that is not devoted to the active elaboration of the emotional experience reduces rumination) [21,22]. Curci and colleagues [3] found that reading negative material increased the challenge for participants to perform the verbal WM task (Experiment 1), whereas watching a negative video clip impaired both verbal and visuospatial performance at the WM task (Experiment 2). They also found that performing a visuospatial task resulted in enhanced rumination only when participants were exposed to verbal emotional material. Taken together, the results of these two studies also demonstrated a strong link between working memory, rumination, and cognitive performance, which is in part influenced by the modality of both the task adopted and the emotional material to be elaborated.

### 1.2. Rumination and Executive Functions

In addition to studies showing a link between rumination and WM, other studies support a relationship between rumination and other core executive functions (EFs) [23,24,25,26,27,28,29]. EFs are higher-level cognitive processes that control and modulate low-level processes to guide individuals in everyday activities [2,30,31,32]. Based on Baddeley’s WM model [33] (but see also Miyake and colleagues [34]), EFs are strictly related to WM. As such, one component of the WM system, the central executive, is the one responsible for the control of high-level cognitive processes such as EFs. In line with the Miyake model [34], three EFs seem to guide human cognition: updating (i.e., the ability to integrate new and relevant information with old information), shifting (i.e., the ability to switch among tasks or information), and inhibition (i.e., the ability to suppress irrelevant information). Although the three EFs work simultaneously, they differ from each other, and they impact different cognitive and social processes, such as memory or theory of mind [31,35,36,37].

Previous studies have hypothesized that rumination is related to EF performance. For example, in 1996, Linville [27] proposed a link between rumination and inhibition, precisely arguing that failure to inhibit information results in an inability to stop and control repetitive thoughts, thus leading to ruminative thinking for long periods of time. Similarly, Koster and collaborators [24] found that people having high traits of ruminative thinking exhibit lower inhibition abilities compared to those with low traits of rumination. They justified this finding by arguing that individuals’ poor inhibition abilities made them prone to develop ruminative thoughts. Results of studies on inhibition are quite consistent with each other [25,26,38,39,40,41,42,43,44]; by contrast, studies on rumination and shifting have been inconclusive in their results. On the one hand, there are studies showing a negative correlation between shifting and rumination [45,46,47,48,49,50,51]; on the other hand, we have studies showing no statistically significant association between shifting and rumination. Likewise, it is unclear whether there is a link between rumination and updating due to a paucity of studies [26,46].

### 1.3. Overview of the Current Study

**Figure 1:**
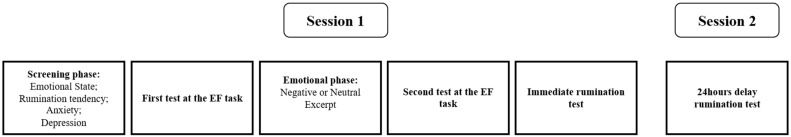
The procedure of Study 1 and Study 2. In Study 1, the EF task measured shifting capacities, while in Study 2, updating abilities.

In two experiments, we wanted to investigate how individuals’ EF abilities would compete with the processing of an emotional experience, thus influencing both short- and long-term emotion regulation of rumination. More precisely, we were interested in extending prior studies by Curci and colleagues [2,3] and understanding whether individual abilities of EFs of shifting and updating (Please note, we did not investigate the inhibition abilities because, as explained in the introduction, studies on this EF and rumination are consistent with each other. Contrarily, this is not the case for the EFs of shifting and updating.) would interact with the negative valence of an excerpt and would affect individuals’ ability to perform EF tasks in immediacy and in their subsequent tendency to ruminate. We intended rumination as an ordinary process subsequent to a negative experience (i.e., no depressive rumination or rumination in clinical population). In both studies, we adopted Curci and colleagues’ [3] procedure and materials with some slight differences. In particular, we asked participants to fill in some screening questionnaires and to perform an EF task. The EF task differed in the two studies as, in Study 1, we were interested in testing individuals’ shifting abilities, while, in Study 2, in assessing individuals’ updating abilities (i.e., test). After this task, in both studies, participants read either a neutral or negative excerpt, and then they repeated the shifting task (Study 1) or the updating task (Study 2) (i.e., retest) a second time. In addition, to have an immediate measure of the ruminative process, we asked participants to reply to the Event Related Rumination Inventory (i.e., immediate). This test was filled in again by participants after 24 h (i.e., 24 h delay). Figure 1 provides an overview of the procedure for the two studies.

We predicted that both EFs of shifting and updating would compete with the emotional elaboration of the negative emotional experience, such that it would affect rumination. Hence, we expected to find an impairment in individuals’ EF task performance (i.e., Study 1: shifting; Study 2: updating) at the retest after the presentation of the negative excerpt [2,26,52,53] (Hp1). In addition, we expected to find higher scores of immediate rumination and prolonged ruminative thoughts in participants who read the negative excerpt compared with participants reading the neutral excerpt [2,3] (immediate rumination: Hp2; 24 h delay rumination: Hp3). Finally, we predicted an interaction effect of individuals’ EF abilities assessed by the test (i.e., Study 1: shifting; Study 2: updating) and the valence of the excerpt to be read (negative vs. neutral) on both immediate and 24 h delay rumination [3], so that the lower individuals’ EF, the higher the frequency of their ruminative thoughts, especially in the negative condition (Hp4).

The Ethical Committee of the Department of Education, Psychology, and Communication of the University of Bari “Aldo Moro” approved both studies. In both studies, participants did not receive any compensation, and they were tested individually.

## 2. Materials and Methods

***Study 1***

### 2.1. Design and Sample

This study adopted a between-subjects design with emotional valence (neutral vs. negative) as a between-subjects variable. The dependent variables in this study were the individuals’ performance at the shifting task and the measures of rumination. Shifting performance was tested on two occasions, i.e., before reading the excerpt and after it (i.e., test and retest). Similarly, rumination was assessed on two occasions, during the first session and 24 h later (i.e., Time).

Using G*Power [54], an a priori power analysis for a 2 × 2 mixed-subjects ANOVA with a power of 0.80 and a medium effect size (f = 0.25) suggested that a number of 34 participants was needed. Hence, we recruited 64 participants using advertisements, but 16 participants were excluded because they did not perform both sessions. Our final sample comprised 48 participants (*M*_age_ = 22.48, *SD* = 3.14, range 18–30; 36 women, 12 men) randomly assigned to one of the emotional conditions (i.e., neutral: *n* = 23, negative: *n* = 25).

### 2.2. Procedure and Measures

This study consisted of two sessions with a delay of 24 h. The first session lasted around 45 min, while the second was 20 min, approximately. Accordingly with Curci and colleagues’ [3] procedure—after providing their consent to participate in this study—participants had to perform different phases for the first session of this study: (a) a screening phase during which they responded to the Positive and Negative Affective Scale-Trait and State (PANAS-T and S) [55], the Ruminative Response Scale (RRS) [56], the State Trait Anxiety Inventory-Form Y (STAI-Y) [57], and the Beck Depression Inventory-II (BDI-II) questionnaires [58] (Please note, these measures were administered to exclude participants who met the criteria for depression and anxiety problems as well as to check any possible differences between groups before the emotion induction (i.e., negative vs. neutral excerpt)), (b) the first shifting task evaluation, (c) an emotion stimulation block during which they were tasked with reading either a neutral or negative narrative, (d) the second shifting task evaluation, (e) the manipulation check phase, (f) the rumination test phase requiring participants to complete the Event Related Rumination Inventory (ERRI) [13]. During the second session, participants were invited to respond to the ERRI questionnaire again.

**Session 1**

***Screening.* The Positive and Negative Affective Scale-Trait and State** (PANAS-T and S) [55]. The questionnaire measures participants’ emotional state across two scales: The positive affect (PA) and the negative affect (NA). Both scales consist of 10 items with a 5-point response (0 = not at all, 4 = completely). The PA scale assesses the positive affective and emotional state, while NA the negative state. In addition, the PANAS-T requires participants to answer the items by thinking about their general emotional state (PA-T Cronbach’s α in the present study = 0.82, NA-T Cronbach’s α in the present study = 0.79), whereas the PANAS-S by reporting their emotional state while there are filling it (PA-S Cronbach’s α in the present study = 0.80, NA-S Cronbach’s α in the present study = 0.85). The scores of each of the ten items of both scales are summed up to obtain the final scores. The higher the PA score, the higher the positive state; similarly, the higher the NA score, the higher the negative emotional state.

**Ruminative Response Scale** (RRS) [56]. The scale consists of twenty-two 4-point items (1 = never; 4 = always) to assess the tendency to think about sad or depressive feelings. It is divided into three subscales: brooding (11 items; Cronbach’s α in the present study = 0.77) evaluates how an individual thinks and re-thinks about the event; reflection (6 items, α di Cronbach’s α in the present study = 0.88) assesses the tendency to ponder about an experience; Depressive Rumination (5 items, Cronbach’s α in the present study = 0.76) refers to the inclination to reflect on events and feelings. Items are summed into each of the three subscales based on the validation score system of the scale.

**State-Trait Anxiety Inventory-Form Y** (STAI-Y) [57]. The inventory consists of 20 items with a 4-point Likert scale (0 = not at all, 4 = completely) and aims to assess feelings of anxiety in the person. The instructions for participants are to reply to the statements by considering their general state. The reliability coefficient of the scale in this study was 0.78. The final score is obtained by summing the scores reported at each item.

**Beck Depression Inventory-II** (BDI-II) [58]. It is a 21-item measure of depression assessing the presence of depressive symptoms in the past two weeks. Participants rate each item on a 4-point Likert scale (e.g., 0 = I do not feel I am worthless, 3 = I feel utterly worthless; range: 0–63). In this study, the reliability coefficient of the scale was 0.83. To calculate the score, the items’ responses are summed up.

***First test at the Shifting task (i.e., test).*** The task is an adaptation of the visuospatial task used in Curci and colleagues’ [3] study. It is a dual task composed of three blocks -after a practice block allowing participants to familiarize themselves with the task of five trials, including from two to six visual elements. Hence, each trial consists of 60 visual elements (i.e., Japanese ideograms). Trials are randomly presented to avoid any expected effect. Each ideogram is presented on one of the four quadrants in which the screen is divided. A fixation point appears for 300 ms, after which follows a blank screen. The participants receive the instructions at the beginning of each trial. The instructions are to memorize the ideograms and their position. Then, after each trial, participants are presented with a black screen and must recognize if the ideograms were the ones watched before. After this recognition task, the participants must also recognize the position where they watched the ideograms. Hence, they are invited to use the mouse to indicate in which quadrant of the screen the ideograms appeared (the stimuli location). However, while doing this second task, we introduced a variant compared to Curci and colleagues’ [3] previous task in order to assess individuals’ shifting abilities. Indeed, we added an auditory stimulus immediately before the beginning of every trial. The additional instruction was to switch the order in which the participants had to remember the location of ideograms. That is, if the participants had the instruction to report the sequence of ideogram positions in the same order as they watched them (i.e., forward order) when they heard the auditory stimulus, they had to report the sequence of position of the remaining ideograms in a reverse order. To make sure to test individuals’ shifting ability instead of the difficulty of remembering positions in a forward or reverse order, we created two versions of the task: One with the primary instruction to start with the forward order and switch to a reverse order once they heard the auditory stimulus (participants = 24) and a second version with the primary instruction to start with a reverse order and switch to the forward order (participants = 24). In both versions, the auditory stimulus was introduced eleven times for block. The score is calculated by considering both the recognition and position tasks. Precisely, the participants can reach a score between 0 and 60 according to how many ideograms and the ideograms positions correctly recognized. In order to ensure our task correctly measured individuals’ shifting abilities, we also administered participants a validated measure assessing the capacity of shifting, i.e., the Plus Minus Task (PMT), and a general measure of working memory, i.e., the Digit Span (DS) task. The scores reported to these tasks and the shifting task were correlated, and we found statistically significant correlations between them, PMT-shifting task Spearman’s r = 0.32, *p* = 0.03, DS-shifting task Spearman’s *r* = 0.28, *p* = 0.05.

***Emotion induction phase.*** Participants had to read either a two-page neutral excerpt or a two-page negative excerpt. As in Curci and colleagues [3], the neutral excerpt corresponded to the instructions and rules of the *Game of the Goose*. By contrast, the negative excerpt was from Muramaki Haruki’s novel *The Wind-Up Bird Chronicle*. The narrative presented a very detailed and faithful description of the torture of a prisoner by Mongolian soldiers.

***Retest at the Shifting task (i.e., retest).*** After reading one of the narratives, participants had to perform the same exact task again immediately before reading either the negative or neutral excerpt (i.e., test).

***Manipulation check.*** To understand whether the excerpt correctly induced the emotional state, participants were asked to describe the content of the excerpt briefly and then to evaluate its emotional impact on a scale of 11 points (0 = not at all, 10 = completely). In addition, they were asked to fill again the PANAS-S [55] (PA-S Cronbach’s α = 0.78, NA-S Cronbach’s α = 0.86).

***First rumination assessment (i.e., immediate).* Event-Related Rumination Inventory** (ERRI) [13]. The questionnaire is a 20-item self-rated measure of intrusive and deliberate rumination. Participants rate the frequency of their ruminative thoughts concerning the excerpt read in the emotional induction phase using a 4-point scale (not at all = 0 to 3 = often). It is possible to derive two scales, each of 10 items: The first scale addresses the frequency of involuntary thoughts and memories (i.e., intrusive; Cronbach’s α in the present study = 0.63), whereas the second measures the frequency of deliberative thoughts (i.e., deliberate; Cronbach’s α in the present study = 0.73). Both final scores are obtained by summing up the scores of each of the ten items. For both scales, a higher value indicates a greater tendency for intrusive or deliberative thoughts.

**Session 2**

***Second rumination assessment (i.e., 24 h delay). Event-Related Rumination Inventory.*** After 24 h from the first session, participants were asked again to fill in the ERRI questionnaire [13], taking into consideration the frequency of ruminative thoughts related to the excerpt read in the prior session (i.e., emotional induction phase). The reliability measures at this test were for the intrusive scale of 0.60 and the deliberate scale = 0.77.

### 2.3. Results

***Screening Analysis***

Three *independent sample t-tests* with emotional valence (neutral vs. negative) as a between-factor were run on brooding, depression, and reflection scores of the RRS in order to verify whether the groups of participants did not differ in their ruminative style prior to the emotional induction. The analyses did not demonstrate any statistically significant differences, brooding, *t*(46) = 1.15, *p* = 0.25, *d* = 0.33; depression, *t*(46) = 1.57, *p* = 0.12, *d* = 0.45, and reflection, *t*(46) = 0.65, *p* = 0.66, *d* = 0.19. The same analyses were conducted on the PANAS-T scores to check the emotional state of participants, as well as on the BDI-II and STAI-Y scores to check differences between groups with regard to depressive and anxiety symptoms, respectively. Overall, none of the analyses demonstrated a statistically significant difference between groups: PA-T, *t*(46) = 0.28, *p* = 0.78, *d* = 0.08; NA-T, *t*(46) = 0.87, *p* = 0.39, *d* = 0.25; BDI-II, *t*(46) = 0.56, *p* = 0.60, *d* = 0.16, and STAI-Y, *t*(46) = 0.78, *p* = 0.44, *d* = 0.23. Descriptives are shown in Table 1.

***Manipulation check***

To check whether the emotional induction worked, a 2 × 2 ANOVA with emotional valence (neutral vs. negative) as a between-factor and pre–post as a within-factor was conducted on PA-S and NA-S scores. Regarding the PA-S score, the analysis showed no statistically significant main effect of emotional valence, *F*(1, 46) = 0.95, *p* = 0.34, *ƞ_p_*^2^ = 0.02, as well as of pre–post, *F*(1, 46) = 5.56, *p* = 0.12, *ƞ_p_*^2^ = 0.11. By contrast, the interaction effect was found to be statistically significant, *F*(1, 46) = 12.37, *p* < 0.001, *ƞ_p_*^2^ = 0.21. Participants in the negative condition reported a lower score after reading the excerpt than before reading the excerpt, *t*(24) = 3.57, *p* < 0.001, *d* = 0.51, *M*_pre_ = 26.63, *SD*_pre_ = 6.29 vs. *M*_post_ = 22.67, *SD*_post_ = 9.41. No statistically significant main effects of emotional valence and pre–post were found also on NA-S, (1, 46) = 0.78, *p* = 0.38, *ƞ_p_*^2^ = 0.02, *F*(1, 46) = 0.24, *p* = 0.63, *ƞ_p_*^2^ = 0.001, respectively. A statistically significant interaction effect was found, *F*(1, 46) = 6.50, *p* = 0.01, *ƞ_p_*^2^ = 0.12, but correct post hoc comparisons did not confirm any statistically significant differences.

Moreover, when we checked how participants evaluated the intensity of the excerpt, we found a statistically significant difference between the group of participants who read the negative excerpt and those who read the neutral one, *Welch’s t*(35) = 6.2, *p* < 0.001, *d* = 1.78. Precisely, participants who read the negative excerpt reported a higher emotional intensity score (*M* = 4.92, *SD* = 3.00) than those who had the neutral excerpt (*M* = 0.76, *SD* = 1.38).

***Analysis of Shifting Performance***

**Figure 2:**
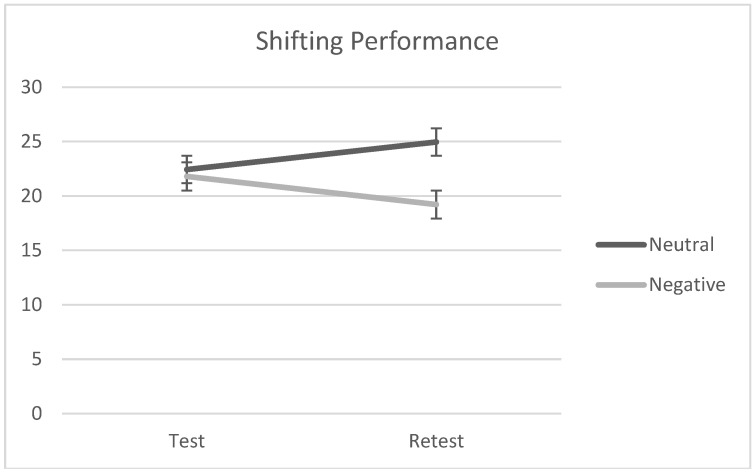
Participants’ performances at the shifting task at test and retest by emotional valence (negative vs. neutral).

To understand whether reading the neutral vs. negative excerpt affected individuals’ performance at the shifting task (Hp1), we ran a 2 × 2 ANOVA with emotional valence (neutral vs. negative) as a between-factor and test–retest as a within-factor on shifting scores. The analysis showed no statistically significant main effects of emotional valence and test–retest, *F*(1, 46) = 1.43, *p* = 0.24, *ƞ_p_*^2^ = 0.03, and *F*(1, 46) = 2.76, *p* = 0.78, *ƞ_p_*^2^ = 0.002, respectively. By contrast, the interaction effect was found to be statistically significant, *F*(1, 46) = 5.56, *p* = 0.02, *ƞ_p_*^2^ = 0.11. Simple effect analyses showed a statistically significant effect of test–retest in the negative condition such that people performed worse at retest than at test, *F*(1, 48) = 5.36, *p* = 0.03, *ƞ_p_*^2^ = 0.11, but the same was not found in the neutral condition, *F*(1, 48) = 1.51, *p* = 0.21, *ƞ_p_*^2^ = 0.02. Descriptive scores are reported in Table 2, but see also Figure 2.

***Analysis of Rumination***

**Figure 3:**
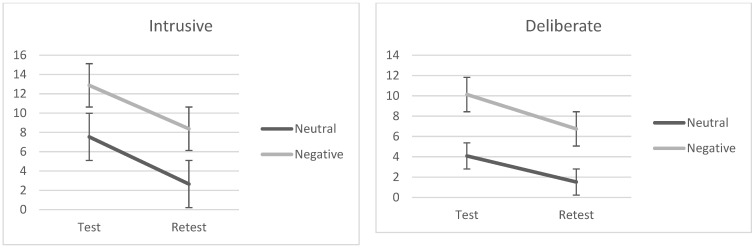
Participants’ performances at ERRI (i.e., intrusive and deliberate scores) at test and retest at Study 1 by emotional valence (negative vs. neutral).

To test our hypotheses 2 and 3, we conducted two 2 × 2 ANOVAs with emotional valence (neutral vs. negative) as a between-factor and time (immediate and 24-h delay) as a within factor on ERRI scores (i.e., intrusive and deliberate). Concerning the analysis of the intrusive score, both main effects of emotional valence and time reached statistical significance, *F*(1, 46) = 8.69, *p* < 0.005, *ƞ_p_*^2^ = 0.16, and *F*(1, 46) = 29.88, *p* < 0.001, *ƞ_p_*^2^ = 0.40, respectively. The intrusive score was lower at the retest than at the test and was higher for participants who had the negative excerpt than those who had the neutral one. The interaction effect was not statistically significant, *F*(1, 46) = 0.05, *p* = 0.83, *ƞ_p_*^2^ = 0.001. Similarly, the analysis of the deliberate score demonstrated that the main effects of emotion valence and time were both statistically significant, *F*(1, 46) = 13.20, *p* < 0.001, *ƞ_p_*^2^ = 0.23 and *F*(1, 46) = 11.23, *p* = 0.002, *ƞ_p_*^2^ = 0.20, respectively. More specifically, the deliberate score was lower at retest than the test and was higher for participants who read the negative excerpt than those who read the neutral one. No statistically significant interaction effect was found, *F*(1, 46) = 0.21, *p* = 0.65, *ƞ_p_*^2^ = 0.005. Table 2 displays descriptive scores, but see also Figure 3.

We further conducted linear regression analyses on ERRI scores to test Hp4. Concerning the ERRI scores at the test, we found no statistical significance of the main effects of shifting performance and emotional valence on the intrusive score, but we found that the interaction effect was statistically significant, *β* = 0.32, *p* < 0.05. Overall, the model fit indices were *R*^2^ = 0.08, *F*(1, 46) = 5.26, *p* < 0.05. By contrast, for the deliberate score, we found no statistically significant main effect of shifting performance and of the interaction effect, but the main effect of emotional valence reached the statistical significance, *β* = 0.41, *p* < 0.01. The model fit indices were *R*^2^ = 0.15, *F*(1, 46) = 9.00, *p* < 0.001. Similarly, regarding the ERRI scores at retest, no statistical significance of the main effects of shifting performance and emotional valence on the intrusive score were detected. The interaction effect was statistically significant, *β* = 0.50, *p* < 0.001. The model fit indices were *R*^2^ = 0.23, *F*(1, 46) = 14.68, *p* < 0.001. Concerning the deliberate score, no statistically significant main effect of shifting performance and interaction effect were found; however, the main effect of emotional valence reached the statistical significance, *β* = 0.44, *p* < 0.001. Overall, the model fit indices were *R*^2^ = 0.17, *F*(1, 46) = 10.67, *p* < 0.01.

### 2.4. Discussion

The findings of Study 1 align with our expectations. In line with Hp1, we found an impairment in individuals’ performance at the shifting task after reading the excerpt only in the group in the negative condition. Hence, we confirmed that the emotional valence of an experience affects an individual’s ability to perform a cognitive task [2,3]. Furthermore, we also found support for Hp2 and Hp3, such that we found higher ruminative thoughts in people who read the negative excerpt than those who received the neutral one [2,3]. Moreover, our results support the assumption that individuals’ shifting abilities predict the persistence of ruminative thoughts for emotional experiences (Hp4). In other words, our data replicated prior studies [3] and further demonstrated that the competition between resources used for the shifting task and resources needed for the post-emotional process of the emotional event resulted in a prolonged rumination. This was found for the specific score intrusive of ERRI, displaying that this relationship affects specific aspects of the tendency to experience repetitive thinking.

***Study 2***

### 2.5. Design and Sample

As in Study 1, Study 2 adopted a between-subject design with emotional valence (neutral vs. negative) as the between-subjects variable. The dependent variables were the same as in Study 1, except for the individual’s performance at the task, which was the updating task instead of the shifting task.

In line with the a priori power analysis conducted for Study 1, the minimum number of participants needed to be included was 34. We thus recruited 55 participants; however, 13 did not complete both sessions; hence, they were excluded, leading to a final sample of 42 participants (*M*_age_ = 22.10, *SD* = 2.80, range 18–28; 32 women, 10 men) distributed to one of the emotional conditions (i.e., neutral: *n* = 21, negative: *n* = 21) in a random fashion.

### 2.6. Procedure and Measures

The procedure and measures used in Study 2 were the same as the ones adopted in Study 1; however—in order to assess individuals’ updating abilities—participants received the updating task instead of the shifting task.

***Updating task.*** As for the shifting task, it is an adaptation of the visuospatial task of Curci and colleagues [3]. Identical to the shifting task, it includes three blocks composed of different trials differing in the number of Japanese ideograms presented. The trial presentation, as well as the general functioning of the task, was the same as the shifting task. The difference regarded the additional introduction of the auditory stimulus and the related instructions. In this version of the task, we added an auditory stimulus during some trial presentations to indicate the ideograms that participants had to remember for the following recognition and position tasks. In other words, participants were instructed to refresh their memory every time they heard an auditory stimulus and to restart the memorization of the ideograms and their positions presented after the auditory stimulus. In addition, to avoid any primacy or recency effect, we introduced the auditory stimuli only for trials having more than three ideograms. Thus, the final score was calculated considering only these trials, and the score participants could obtain ranged from 0 to 48. To check the validity of our task to measure individuals’ abilities of updating, we administered participants a validated measure of updating, i.e., the phonemic fluency (PF), and a general measure of working memory, i.e., the Digit Span (DS) task. Correlational analyses demonstrated a significant association of the tasks, PF-updating task Spearman’s *r* = 0.40, *p* = 0.03, DS-updating task Spearman’s *r* = 0.33, *p* = 0.05.

### 2.7. Results

***Screening Analysis***

To ensure no difference occurred between groups with regard to ruminative style, a set of *independent sample t-tests* with emotional valence (neutral vs. negative) as a between-factor was run on brooding, depression, and reflection scores. No statistical significance was detected for the brooding, depression, and reflection scores, *t*(40) = 1.00, *p* = 0.32, *d* = 0.31, *t*(40) = 0.18, *p =* 0.86, *d* = 0.06, *t*(40) = 1.61, *p* = 0.12, *d* = 0.50, respectively. Similarly, *independent sample t-tests* were run on PANAS-T, the BDI-II, and STAI-Y scores to check differences regarding individuals’ emotional state, and depressive and anxiety symptoms. The analyses did not show any difference statistically significant between groups: PA-T, *t*(40) = 0.55, *p* = 0.59, *d* = 0.17; NA-T, *t*(40) = 0.61, *p* = 0.55, *d* = 0.19; BDI-II, *t*(40) = 0.44, *p* = 0.66, *d* = 0.14, and STAI-Y, *t*(40) = 0.26, *p* = 0.80, *d* = 0.08.

***Manipulation check***

To check whether the emotional induction worked, a 2 × 2 ANOVA with emotional valence (neutral vs. negative) as a between-factor and pre–post as a within-factor was conducted on PA-S and NA-S scores. Regarding the PA-S score, the analysis showed no statistically significant main effects of emotional valence and pre–post, *F*(1, 40) = 0.02, *p* = 0.96, *ƞ_p_*^2^ = 0.001, *F*(1, 40) = 3.13, *p* = 0.08, *ƞ_p_*^2^ = 0.07 as well as of the interaction effect, *F*(1, 40) = 1.45, *p* = 0.24, *ƞ_p_*^2^ = 0.04. No statistically significant main effects of emotional valence and pre–post were found on NA-S, *F*(1, 40) = 3.29, *p* = 0.08, *ƞ_p_*^2^ = 0.08, *F*(1, 40) = 0.16, *p* = 0.69, *ƞ_p_*^2^ = 0.004, respectively. By contrast, the interaction effect was found to be statistically significant, *F*(1, 40) = 20.69, *p* < 0.001, *ƞ_p_*^2^ = 0.34. Participants in the negative condition reported a higher score after reading the excerpt than before reading the excerpt, *t*(21) = 2.37, *p* = 0.03, *d* = 0.51, *M*_pre_ = 6.55, *SD*_pre_ = 8.13 vs. *M*_post_ = 9.59, *SD*_post_ = 8.17.

Furthermore, participants’ evaluation of the emotional intensity of the excerpts statistically differed between those who read the negative excerpt and those who read the neutral one, *Welch’s t*(36) = 3.72, *p* < 0.001, *d* = 1.14, such that participants who read the negative excerpt reported a higher emotional intensity score (*M* = 4.18, *SD* = 3.13) than those who had the neutral excerpt (*M* = 1.21, *SD* = 1.93).

***Analysis of Updating Performance***

**Figure 4:**
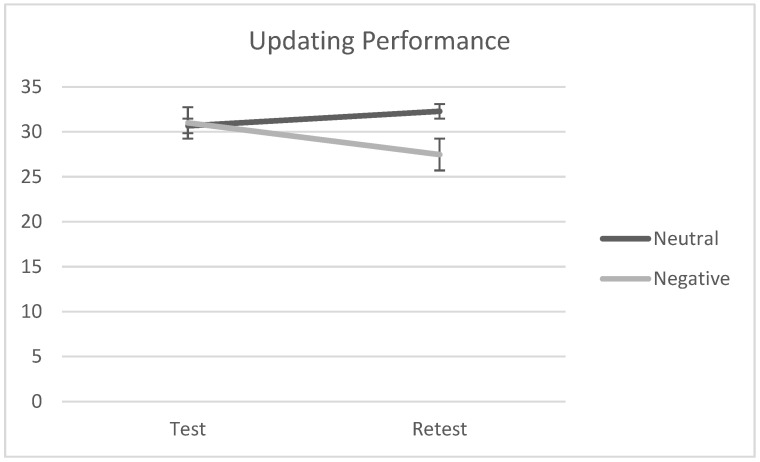
Participants’ performances at the updating task at test and retest by emotional valence (negative vs. neutral).

A 2 × 2 ANOVA with emotional valence (neutral vs. negative) as a between-factor and test–retest as a within-factor was conducted to check the impact of the emotional induction on individuals’ performance at the updating task (Hp 1). The analysis demonstrated neither statistical significance for the main effects of emotional valence and test–retest nor for the interaction effect, *F*(1, 40) = 0.41, *p* = 0.53, *ƞ_p_*^2^ = 0.10, *F*(1, 40) = 0.29, *p* = 0.59, *ƞ_p_*^2^ = 0.007, and *F*(1, 40) = 2.14, *p* = 0.15, *ƞ_p_*^2^ = 0.05, respectively. Descriptive scores are reported in Table 3, but see also Figure 4.

***Analysis of Rumination***

**Figure 5:**
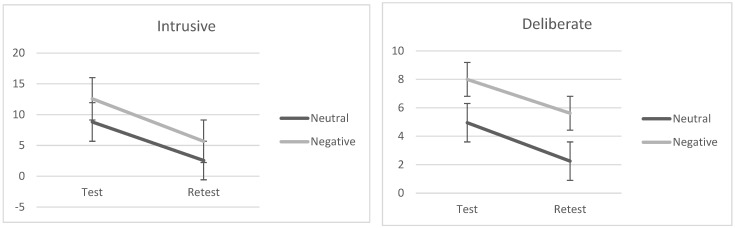
Participants’ performances at ERRI (i.e., intrusive and deliberate scores) at test and retest at Study 2 by emotional valence (negative vs. neutral).

Two 2 × 2 ANOVAs with emotional valence (neutral vs. negative) as a between-factor and time (immediate and 24 h delay) as a within-factor on ERRI scores (i.e., intrusive and deliberate) were carried out to test hypotheses 2 and 3. The analysis on the intrusive score showed a statistically significant main effect of time, *F*(1, 40) = 38.00, *p* < 0.001, *ƞ_p_*^2^ = 0.48, such that individuals reported a lower score at 24-h delay than in the immediacy. Similarly, the main effect of emotional valence was significant *F*(1, 40) = 5.12, *p* = 0.03, *ƞ_p_*^2^ = 0.12; specifically, participants in the negative condition reported a higher score than those in the neutral condition. No statistical significance was found for the interaction effect, *F*(1, 40) = 3.32, *p* = 0.13, *ƞ_p_*^2^ = 0.003. The same pattern of results was found for the deliberate score, such that we found a statistically significant main effect of time, *F*(1, 40) = 5.75, *p* = 0.02, *ƞ_p_*^2^ = 0.13 and emotional valence, *F*(1, 40) = 3.28, *p* = 0.04, *ƞ_p_*^2^ = 0.08. No statistical effect was found for the interaction effect, *F*(1, 40) = 0.02, *p* = 0.88, *ƞ_p_*^2^ = 0.001. Descriptive scores are shown in Table 4, but see also Figure 5.

In order to test Hp4, we conducted linear regression analyses on ERRI scores on updating performance, emotional valence, and updating by emotional valence. These analyses showed no significant effect (all *p_s_* > 0.05).

### 2.8. Discussion

The results of Study 2 are not completely in accordance with our expectations. As a matter of fact, we did not find any difference between groups with regard to their performance at the updating task. This means that, contrarily to Hp1, the negative valence of the excerpt did not affect people’s ability to complete the cognitive task. By contrast, Hp2 and Hp3 were sustained by our findings. We indeed found an effect of time on the ERRI scores, such that people reported lower scores 24 h later than at session 1 (i.e., immediate). Moreover, we found that participants reported a higher level of ruminative thought when they read the negative excerpt than when assigned to the neutral condition, in line with prior studies showing that a negative experience leads to rumination [3]. Finally, our data showed no effect of individuals’ updating abilities on the persistence of rumination processes (Hp4). This suggests that these specific EF abilities are not implicated in the competition for resources; hence, they do not contribute to prolonging post-emotional rumination.

## 3. General Discussion

In the present two studies, we aimed to extend prior studies by Curci and colleagues [3] by investigating the relationship between individuals’ cognitive abilities and rumination in the short and long term, taking into account two specific components of individuals’ executive functioning, i.e., shifting and updating abilities. In both studies, we expected to find in line with prior studies [2,3], and due to the strong link between working memory and shifting and updating [34,59,60], an impairment of people’s performance at these cognitive tasks and a higher rumination in people who received the negative excerpt than in people who received the neutral one. We also expected a prolonged effect of rumination in people who read the negative excerpt. Finally, we predicted to find an effect of individuals’ shifting and updating abilities on ruminative thoughts experienced in the long term. Collectively, our results did not completely show the expected pattern of results. Indeed, in Study 1, data supported all our predictions. Differently, in Study 2, we found that rumination was higher in those who read the negative event, but we also found that, overall, it decreased over time regardless of the emotional induction in participants.

In line with the theoretical account relying on the WM model [33], our findings on individuals’ performance at the cognitive task support. This theory posits that WM has a limited capacity, and as such, when people experience upsetting events, the cognitive representations formed after the experience compete with cognitive resources normally devoted to other cognitive tasks [61]. Similarly to prior findings [2], individuals reported poor performance at the shifting task after they read the negative excerpt. Hence, these results not only replicated prior findings but shed new light on the understanding of the specific components of WM implicated in this relationship. As a matter of fact, we found a depleted cognitive performance after the emotional experience only at the shifting task. No statistically significant effect was found for the updating task, although people who read the negative excerpt seemed to ruminate less after being exposed to that material. Although the EF tasks are not directly comparable, if we consider the average levels of the EF performance reported by participants for the shifting and updating tasks, we notice that participants reported higher scores for the updating task than for the shifting task. It could be, therefore, that participants found the execution of the updating task as compared with the shifting one. Thus, performing the updating task seemed to require a moderate cognitive effort such that participants could perform it even after the emotional experience.

With regards to rumination, in both studies, in line with our expectations, people reported higher scores of rumination when they read the negative excerpt than those who received the neutral one. This result was found for both scales of the ERRI questionnaire and is in line with prior literature showing that negative emotional experiences elicit ruminative thinking [2,3]. In addition, in both studies, we also found that rumination decreased over time regardless of having been exposed to a negative event or a neutral one. A possible explanation for these findings can be found in a prior hypothesis that rumination persists over time when the emotional experience and concurrent task share the same modality [19,20,62,63]. Based on this hypothesis, when people are exposed to verbal emotional material and perform a verbal cognitive task, their ruminative thoughts persist over time. By contrast, when the modality of the cognitive task differs from the emotional experience, rumination will be less lasting. Although in our studies, we did not want to study whether task congruency affects rumination specifically, we can speculate that we found a less prolonged rumination because the modality of the emotional experience differed from the one of the cognitive tasks.

Noteworthy, these results need to be considered in light of the analyses aiming to verify a possible competition of resources between the cognitive task and the emotional processing of the experience. Interestingly, we found support for the hypothesis that the specific EF of shifting plays a role in this competition while updating does not intervene. In fact, our results demonstrated an interaction effect between the emotional valence of the excerpt and individuals’ shifting abilities on rumination after 24 h from the experience. This underlines that—although overall rumination decreased over time—shifting abilities can predict people’s tendency to persist in ruminative thoughts after an upsetting event. These findings are congruent with Curci and colleagues’ [2,3] studies, and they are also in line with studies showing a correlation between shifting and rumination [45,46,47,48,49,50,51]. Yet, findings on updating abilities add to prior literature testing a possible involvement of this EF in rumination. So far, a few studies on the link between updating and rumination have achieved contrasting results [26,46]. Our data seem to hint at the idea that updating is not directly involved in such a link. Again, the lack of significance in Study 2 could depend on the possibility that individuals employed a low amount of cognitive resources for the execution of the updating task and, therefore, the competition of resources between the post-emotional processing and the cognitive task was lower. Moreover, another possible speculation is related to the type of updating measure used in our study. Indeed, in real experiences, in order to reduce or stop ruminative thoughts concerning a negative event, individuals have to operate an update of their emotional and event-related thoughts with others less emotional. In our study, we did not directly assess this capacity; instead, we used a pure measure of executive functioning. This is an important aspect to consider while reading our results, and it could be a relevant issue for future investigation.

Together with the just mentioned aspect, a second limitation of our studies was that we did not include a measure of intrusions (i.e., not deliberate sensory and visual images of the emotional event). Prior studies have underlined that intrusions and rumination are affected differently by the individuals’ availability of WM resources [3]. Hence, it would be interesting to understand whether and how the specific individuals’ resources of shifting and updating affect intrusions. Finally, another caveat of our studies was that we included in our design only a negative event. Nevertheless, rumination can also occur for positive events, i.e., positive rumination [10]. Consequently, future researchers should consider including in their studies an emotionally positive stimulus.

In sum, our results extended prior studies on executive processing and rumination after an emotional experience. We were interested in disentangling the role of two specific executive functions in this relationship. In line with prior studies, we showed that even negative experiences can have a cognitive cost in the short and long term. This cognitive cost seems related to specific individuals’ executive functioning abilities (i.e., shifting). Therefore, we provided novel and relevant insights on the comprehension of the interplay between the cognitive process and the emotion regulation process of rumination.

## Figures and Tables

**Table 1:** Mean proportions of the screening measures reported by participants at Study 1 by emotional valence (neutral vs. negative). Standard deviations and 95% CI are shown between parentheses.

	Neutral	Negative
**Brooding**	22.74 (6.94), 95% CI [19.74, 25.74]	20.72 (6.73), 95% CI [18.61, 22.83]
**Depression**	12.09 (4.36), 95% CI [10.20, 13.97]	10.32 (3.44), 95% CI [8.90, 11.74]
**Reflection**	9.70 (3.76), 95% CI [8.07, 11.37]	9.08 (2.68), 95% CI [7.98, 10.18]
**PA-T**	28.04 (5.20), 95% CI [25.79, 30.29]	28.60 (8.05), 95% CI [25.28, 31.92]
**NA-T**	8.87 (8.45), 95% CI [5.22, 12.52]	10.80 (6.86), 95% CI [7.97, 13.63]
**BDI-II**	9.87 (9.75), 95% CI [5.65, 14.09]	8.60 (6.47), 95% CI [5.93, 11.27]
**STAI-Y**	43.65 (11.62), 95% CI [38.63, 48.68]	41.44 (7.76), 95% CI [38.24, 44.64]

**Table 2:** Mean proportions of the measures at the shifting task and ERRI questionnaire reported by participants at Study 1 by emotional valence (neutral vs. negative) and time (test and retest) and follow-up (immediate and 24-h delay). Standard deviations and 95% CI are shown between parentheses.

	Neutral		Negative	
	Test	Retest	Test	Retest
**Shifting Performance**	22.43 (11.07), 95% CI [17.65, 27.22]	24.96 (10.84), 95% CI [20.27, 29.64]	21.80 (10.42), 95% CI [17.5, 26.10]	19.21 (11.31), 95% CI [14.43, 23.99]
	**Immediate**	**24 h delay**	**Immediate**	**24 h delay**
**ERRI-Intrusive**	7.54 (6.28), 95% CI [4.81, 10.24]	2.65 (4.21), 95% CI [0.83, 4.47]	12.88 (9.46), 95% CI [8.88, 16.87]	8.38 (7.20), 95% CI [5.33, 11.42]
**ERRI-Deliberate**	4.09 (5.20), 95% CI [1.84, 6.33]	1.52 (2.43), 95% CI [0.47, 2.57]	10.13 (7.87), 95% CI [6.80, 13.45]	6.75 (7.30), 95% CI [3.67, 9.83]

**Table 3:** Mean proportions of the screening measures reported by participants at Study 2 by emotional valence (neutral vs. negative). Standard deviations and 95% CI are shown between parentheses.

	Neutral	Negative
**Brooding**	23.33 (6.25), 95% CI [20.49, 26.18]	21.33 (6.71), 95% CI [18.28, 24.39]
**Depression**	12.10 (3.82), 95% CI [10.36, 13.83]	11.86 (4.61), 95% CI [9.76, 13.95]
**Reflection**	10.52 (3.83), 95% CI [8.78, 12.27]	8.86 (2.82), 95% CI [7.58, 10.14]
**PA-T**	28.57 (5.73), 95% CI [25.96, 31.18]	27.62 (5.50), 95% CI [25.12, 30.12]
**NA-T**	10.00 (5.68), 95% CI [7.42, 12.58]	8.57 (9.15), 95% CI [4.41, 12.73]
**BDI-II**	7.43 (6.54), 95% CI [4.45, 10.41]	8.38 (7.72), 95% CI [5.00, 11.76]
**STAI-Y**	43.19 (8.20), 95% CI [39.46, 46.92]	44.05 (12.78), 95% CI [38.23, 49.87]

**Table 4:** Mean proportions of the measures at the updating task and ERRI questionnaire reported by participants at Study 2 by emotional valence (neutral vs. negative) and Time (test and retest) and follow-up (immediate and 24-h delay). Standard deviations and 95% CI are shown between parentheses.

	Neutral		Negative	
	Test	Retest	Test	Retest
**Updating Performance**	30.67 (11.20), 95% CI [25.57, 35.76]	32.29 (12.98), 95% CI [26.38, 38.19]	31.00 (13.61), 95% CI [24.81, 37.19]	27.48 (12.71), 95% CI [21.69, 33.26]
	**Immediate**	**24-h delay**	**Immediate**	**24-h delay**
**ERRI-Intrusive**	8.81 (5.82), 95% CI [6.16, 11.46]	2.55 (4.05), 95% CI [0.66, 4.44]	12.57 (7.66), 95% CI [9.09, 16.06]	5.67 (6.11), 95% CI [2.89, 8.45]
**ERRI-Deliberate**	4.95 (5.47), 95% CI [2.46, 7.44]	2.25 (6.03), 95% CI [0.57, 5.07]	8.00 (7.41), 95% CI [4.61, 11.39]	5.62 (7.10), 95% CI [2.39, 8.85]

## Data Availability

Authors are willing to share their data under request.
